# The pre-amputation pain and the postoperative deafferentation are the risk factors of phantom limb pain: a clinical survey in a sample of Chinese population

**DOI:** 10.1186/s12871-017-0359-6

**Published:** 2017-05-26

**Authors:** Yan Yin, Lan Zhang, Hong Xiao, Chuan-Bing Wen, Yue-E Dai, Guang Yang, Yun-Xia Zuo, Jin Liu

**Affiliations:** 1 Department of Pain management, West China Hospital, Sichuan University, Chengdu, Sichuan 610041 People’s Republic of China; 2 Department of Anesthesiology, Sichuan Orthopedics Hospital, Chengdu, Sichuan 610041 China; 3 Department of Anesthesiology, Sichuan Academy of Medical Sciences & Sichuan provincial People’s Hospital, Chengdu, Sichuan 610000 China; 4 Department of Anesthesiology, West China Hospital, Sichuan University, Chengdu, Sichuan 610041 China

**Keywords:** Phantom limb pain, Pre-amputation pain, Postoperative deafferentation, Risk factors

## Abstract

**Background:**

To provide an overview of phantom limb pain (PLP) in China. This includes the prevalence of PLP and possible risk factors.

**Methods:**

In a retrospective study, telephone interviews were conducted with 391 amputation patients who underwent extremity amputations at a tertiary hospital in China.

**Results:**

PLP was found in 29% of the amputees. Pre-amputation pain (OR = 10.4, *P* = 0.002) and postoperative analgesia (OR = 4.9, *P* = 0.008) were identified as high-risk factors for PLP. 82.1% of PLP patients experienced pre-amputation pain. The average pain intensity of PLP was 5.1 ± 2.2, with 31.9% having severe intensity. The effects of PLP on the quality of the PLP patients were as follows: 7.8% of the patients had to limit their daily life and 29.0% of the patients had to limit their social activities. 17.3 and 25.7% of patients experienced depression and sleeping disorder respectively, while 18.9% had loss of interest and even 16.1% of PLP patients had attempted suicide. No effective treatments were found in 78.9% of these patients.

**Conclusions:**

PLP has markedly affected the lives of patients. Pre-amputation pain and postoperative epidural analgesia might be risk factors for the phantom limb pain after amputation. Prevention of pre-amputation pain and sudden post-amputation deafferentation should be recommended to the amputees.

## Background

The term ‘phantom limb’ was coined in 1866 by Silas Weir Mitchell, who described phantoms as ghostly replicas of the lost limb, some of which are painful (i.e. phantom limb pain) and others are not (i.e. phantom limb sensations (PLS)) [1, 2]. Phantom limb pain (PLP) has been defined as painful sensations perceived in the missing body part [1]. Although reports on the incidence of PLP among amputee patients is highly variable among and ranges from 2% ~ 98% [2–4], a preponderance of studies report the incidence to be in the 50 ~ 80% range [5–11]. Severe PLP is reported as 5% ~ 10% [2, 3]. Pain after limb amputation is a common sequela that often becomes chronic, as well as limiting quality of life and functional capacity [12, 13]. Those who experience PLP display more indecisiveness, suicidal ideation, and other thoughts of self-harm. Severity of PLP is directly related to such psychological ill effects.

Although diagnosis is uncomplicated, the etiology of PLP, especially risk factors are elusive, and correspondingly, no consensus has emerged on its prophylaxis and management [14, 15]. Moreover, the extant of research on PLP has occurred in the West. However, to our knowledge, there is a paucity of research on the etiology, prevalence, and the consequences on health services of PLP in China. This is a conspicuous shortcoming in the literature because, with a population of more than 1.3 billion people, China composes 19% of humanity. It might be different in the development of PLP between West and Asian for genetics and racial reasons. The culture and medical environment may also play important roles in this disparity. For example, tolerance to pain is considered a virtue in China. And postoperative pain is considered normal and is frequently neglected by patients and physicians. Many surveys conducted in China showed misunderstanding about analgesics and poor management of acute or chronic pain [16–18]. This survey, conducted in China, was designed mainly to investigate the prevalence of PLP and possible risk factors.

## Methods

This study was conducted at West China Hospital, which is an affiliate of Sichuan University. The IRB of West-China Hospital of Sichuan University approved the study.

Patients who underwent amputation from January of 2002 to December of 2013 at West China Hospital (Chengdu, Sichuan Province) were enrolled in this survey. The total number of patients who underwent amputation was 583. Patients were excluded if they had died or they were untraceable, confused, unable to communicate in the telephone interview, or refused to participate. Telephone interviews were conducted with 391 amputation patients, which were conducted by trained and supervised study personnel (research study assistants). Consent prior to the telephone interview was obtained. A random sample of the respondents (*n* = 60) was re-interviewed 7 to 10 days after the initial interview, by an independent interviewer as a reliability check. A total of 10 key questions were repeated.

The patients were telephone interviewed using a phantom limb pain questionnaire that included questions on diverse aspects of PLP. Interviewers were allowed to explain questions if respondents did not understand them. The interview schedule comprised the following five modules: 1) basic demographic variables (gender, age, address, educational level, and occupation) and cause of the amputation; 2) the presence of PLP and when it started, pain intensity, pain description (including total number of words and sensory/affective components), the number of attacks a day, and aggravating/relieving factors; 3) the effective pain treatment and its cost; 4) the impact of the PLP on the patients’ daily life, psychosocial abilities such as work and social abilities and the impact on their mental status; 5) the anesthesia technique used for the amputation; the postoperative analgesia, if there was a preemptive analgesia, and whether they accepted radiotherapy or chemotherapy. The information of modules 1 and 5 was confirmed by reviewing the patient’s medical records. All patients were asked about the presence of PLP and when it started. If PLP was present, the other questions were completed. If PLP was not present, questions about the amputation such as if they had pre-amputation pain, postoperative analgesia, and the effect of the amputation on their life were asked instead.

The intensity of pain was measured by using the pain intensity numeric rate scale (NRS, from 0 = no pain to 10 = worst pain imaginable). Respondents were asked to assess a number along the scale to indicate their most serious level of pain after amputation operation (“How intense was your worst pain?”). The NRS scores were collapsed into mild, moderate, and severe categories of intensity by dividing the pain intensity numeric rate scale as:1 to 3 was mild, 4 ~ 6 was moderate, and 7 ~ 10 was severe.

The consequences of PLP and amputation were described by assessments of the limitation on activities of daily life, social activities and work abilities, employment, expression, downhearted feelings (lack of mental well-being), sleep disturbance, and suicide ideations. PLP as well as amputation would affect the quality of the patients’ life, the difference of the limitation on activities of daily life, social activities and work abilities, employment, expression, downhearted feelings (lack of mental well-being), sleep disturbance, and suicide ideations between the PLP and PLP free amputee were thought to be the net effect of PLP on the quality of PLP patients.

On reliability testing, intraclass correlation (ICC) was used for numerical data between the first and second interviews. For categorical data, kappa statistics were presented. Descriptive statistics, primarily percentages and averages, were used to describe the participants and their experiences of phantom sensations and phantom pain. This was mainly descriptive, using SPSS version 19. Percentages of each phenomenon were calculated. The impact on daily life activities, psychosocial abilities such as work and social abilities and the impact on their mental status in PLP and PLP free patients were compared by *x* ^2^ tests. The correlation of PLP with gender, age, employment status, occupation, and educational level, disease before amputation, the pre-amputation pain and the anesthesia of the amputation were conducted by correlation analysis. Logistic regression was used to identify major risk factors of the PLP.

## Results

An independent interviewer who was blind to the responses from the 10 same key item-questions in the first interview performed a reliability check on 60 respondents. This showed that the information from the survey was reliable, with ICC coefficients ranging from 0.85 to 0.99 while the kappa coefficients ranged from 0.75 to 1.

Of the 391 patients with amputations recruited for the study, one hundred and thirteen (113) patients (29.0%) reported that they experienced PLP. The majority of the PLP patients (77 patients, 68.3%) were male. The ages of the PLP patients ranged from 21 to 81 years old (39 ± 14.9 years), most of whom (70%) ranged from 30 to 59 years old. Nearly 78.4% of the PLP patients reported an education of senior school or below. The most common level of amputation was transfemoral (above knee; 44.4%), with transtibial (below knee; 36.1%) being the next most common. Upper limb amputation (20.8%) was the third most common type of amputation. Trauma including car accident, work injury and earthquake accounted for 60.5% of the amputations, with chronic diseases, including vascular disease accounting for 21.1% and cancer for 18.4% in this sample. In the patients with PLP, the average pain intensity was 5.1 ± 2.2, with 31.9% having severe intensity. 82.1% PLP patients suffered from the pre-amputation pain. Additionally two thirds (66.7%) of those with PLP patients received postoperative analgesia. Routes of analgesic administration for these PLP patients were oral for 35.6% of the patients, intramuscular for 25.7%, intravenous for 33.3% and epidural for 4.4% of the patients. And the dominant medicines used in postoperative analgesia were pethidine, morphine, tramadol and epidural morphine and ropivacaine.

In a multiple logistic regression model including all general socio-demographic characteristics and associated factors, pre-amputation pain and postoperative analgesia were consistently associated with PLP. Patients with pre-amputation pain had a higher prevalence of PLP (OR = 10.4, *P* = 0.002) compared with pre-amputation pain free patients. The use of postoperative analgesia was identified as a high-risk factor (OR = 4.9, *P* = 0.008) of PLP, but no statistically significant difference was found amongst the patients between the different routes of administration of analgesia (oral, intravenous, intramuscular, or epidural). However, taking oral analgesia as the baseline (OR = 1), postoperative epidural analgesia had a higher OR value than intravenous and intramuscular analgesia (OR = 403868716.1, 0.4 and 0.5, respectively).

Of the 29% of patients (113 patients from the total sample of 391) who reported to have PLP, 83.6% of them had to limit their work, 76.1% felt it affected their daily life and 80.6% reported difficulties with social activities. Moreover, 38.8% of the PLP patients experienced dispirited, 46.3% of them had sleeping disorder, and 26.9% of them lost of interest because of the PLP in these 113 interviewed patients. Importantly, 20.9% of the PLP patients attempted suicide. Of the 278 patients who did not have PLP, 58.0% of them had to limit their work, 68.3 and 51.6% of them reported their daily life activities and social activities limited respectively because of the amputation. 44.8% of them reported they lost their jobs due to the amputation. Moreover, 21.5% of them had depression, 20.6% of them had sleeping disorder, and 8% of them had loss of interest. Only 4.8% of them reported they ever had a suicide attempt. After subtracted the influence of amputation in the PLP patients, the effects of PLP on the quality of their lives are as follows: 7.8% of the patients had to limit their daily life and 29.0% of the patients had to limit their social activities and work. 17.3 and 25.7% of patients experienced depression and sleeping disorder respectively, while 18.9% had loss of interest and even 16.1% of PLP patients had attempted suicide (Table 1).

**Table 1:** The consequences of PLP and amputation

	PLP patients (%) *N* = 113	PLP free patients (%) *N* = 278	*p*-values	Difference of PLP and PLP free patients (%)
Work limited	83.6	58.0	0.002	25.6
Lost jobs	65.7	44.8	0.033	20.9
Daily life activities limited	76.1	68.3	0.680	7.8
Social activities limited	80.6	51.6	0.008	29.0
Depression	38.8	21.5	0.037	17.3
Sleeping disorder	46.3	20.6	0.017	25.7
Lost interest	26.9	8	0.024	18.9
Suicide attempt	20.9	4.8	0.004	16.1

The consequence of PLP (phantom limb pain) and amputation on the lives of patients showed in this table. Difference of PLP and PLP free patients = the ratio of the work limited, lost jobs, etc. in PLP patients-the ratio of the work limited, lost jobs, etc. in PLP free patients

In the sample which reported PLP, nearly 80% (78.9%) of them reported that they had no effective treatments for PLP and had to endure pain, while only 21.1% of them reported that they got the relieve after treatments. 69.2% PLP patients only spent below 50 Chinese Yuan (CNY) and 23.1% spent 100 to 300 CNY every month on their treatment for PLP. And another 7.7% patients spent over 2000 CNY monthly to manage their pain.

## Discussion

The purpose of this study was to examine the possible etiology of PLP, as well as determine any risk factors of PLP and the prevalence in a sample of Chinese population. The main results demonstrated that patients with pre-amputation pain in the affected limb and the postoperative analgesia were at great risk for PLP. Patients with pre-amputation pain and who received postoperative analgesia had almost ten times and five times higher prevalence of PLP respectively, compared with pre-amputation pain and postoperative analgesia free patients.

Ten to Fifty percent of patients with postsurgical pain develop chronic pain, which is the primary predictor of patients’ dissatisfaction [19]. The prevalence of chronic postsurgical pain may vary depending on the type of surgery and the amputation is the most common surgery related to persistent postsurgical pain which reported in 50 to 80% patients [5–11]. Preoperative pain, nerve injury, severity of the immediate postoperative pain and opioid consumption are factors associated with increased risk of chronic postsurgical pain [20, 21]. From our investigation, multiple presumed factors for chronic postsurgical pain were examined. All general socio-demographic characteristics such as gender, age, occupation, and educational levels were not associated with the PLP. Furthermore, other multiple characteristics such as chronic disease before amputation, the operative anesthesia for the amputation, and whether or not the subjects accepted radiotherapy or chemotherapy were not associated with the PLP. The results demonstrated that both pre-amputation pain and postoperative analgesia are high risk factors for PLP.

PLP has been shown to be more frequent in patients with pre-amputation pain [22], while in our survey, 80.1% of the PLP subjects reported that they experienced pre-amputation pain in the phantom limb. Amputees due to congenital limb deficiencies or had the amputation during their childhood did not frequently suffer from PLP [23]. Amputees who did not use a prosthesis or used a cosmetic prosthesis had a tendency to suffer from PLP compared with those who used a myoelectric prosthesis [24]. Although the theory of whether or not preemptive analgesia prevent PLP is still controversial, three studies showed a significant reduction in the incidence of PLP, with the preemptive administration of epidural bupivacaine, opioids or clonidine [25–27]. The evidence in our cohort as well as other studies indicated the role of preemptive analgesia in preventing PLP. Current evidence is not in strong support of any one anesthetic technique of postoperative pain control is likely to provide a greater impact on preventing PLP [28–31]. Moreover, we found that patients who accepted postoperative analgesia were had almost a five times higher risk to experience PLP after the amputation, compared with the patients who did not have postoperative analgesia. The exact cause of this founding is not clear. The reason may be due to the fact that these individuals needed more analgesia were probably suffering from worse postoperative pain, which by itself put them at risk for PLP. Another possible mechanism might be that the sudden deafferentation of the amputated extremities advocated the PLP, since postoperative epidural analgesia had a higher OR value than oral, intravenous and intramuscular analgesia (OR = 403868716.1, 1.0, 0.4 and 0.5, respectively). Our findings suggest that the pre-amputation pain and postoperative pain or the sudden deafferentation of the amputated extremities might be possible causes of PLP. Further larger, randomized, controlled clinic studies are required to better determine whether postoperative pain and different analgesia methods can affect the incidence of PLP.

The other purpose of this survey was to investigate the prevalence of PLP in this sample of Chinese population. Phantom limb pain is a common phenomenon in amputees. Most studies report the prevalence of PLP as ranging from 50 to 80% [5–11], while the prevalence in our survey was 29%. However trauma was the cause for 60.5% of the amputations in this study, which is different from other studies which had vascular disease and tumor as the main causes for amputation [3, 32]. When looked individually, the prevalence of PLP in our survey for trauma patients was 26% as compared to 32% for vascular disease and tumor patients. Therefore the difference in the cause of the amputation is not a possible reason for the lower prevalence. The possible causes for this difference between our survey and the literature might be: First, our population was Chinese, while most previous studies had largely Caucasian populations. Caucasians have been shown to have higher levels of PLP when compared to non-Caucasians, a phenomenon that may be associated with a variety of biological, social and psychological mechanisms [33–35]. Second, our study is retrospective, and thus relies upon the patient’s memory. Some patients might forget the mild PLP after amputation.

Studies suggest that work and social abilities are limited in PLP patients and that evidence of clinical depression were observed in these patients. Sherman and Arena [36] found that 33.5 and 44.8% of PLP patients reported that their work and social abilities were limited respectively. Eighty-two percent of amputees had a sleeping disorder and 45% of them had limited activities of daily life. This data was based only on the reports of PLP patients. However, are the limitations on work and social abilities and upon the activities of daily life caused by the PLP or just the amputation itself? In this survey, these activities were investigated in both PLP patients and PLP-free amputees, and it was shown that work and social abilities as well as activities of daily life were more limited in PLP patients. Furthermore, if the influence of amputation is subtracted, the ratios of the limitations on work and social abilities as well as disturbance in sleep are lower than those reported by Sherman and Arena. These ratios were calculated by deducting the ratios of PLP free amputees from the ratios of PLP patients, which is called “the net consequences of PLP”. Thus the net consequences of PLP may stand by the influence of PLP on the patients directly and objectively.

Although this survey shows that PLP really influences the patients’ work and social ability and daily life, indicating that these individuals need of early, intensive pain interventions. This study also shows that the treatment of PLP was poor in these patients. 78.9% of them reported that they were not on effective treatments for the PLP, and most of them (69.2%) only spent less than 50 CNY(almost 7 $US) every month on the PLP. This is partly explained by the mechanism of PLP being unclear thereby calling for more research, and also suggests that PLP has been neglected by the health service of Chinese society.

The major limitation of this study is that it was retrospective and the prevalence was assessed by a single phone interview, and relied upon the amputees’ memory. Thus, there might be some recall bias and the prevalence rate of PLP reported in this study might be underestimated. Future research could overcome this problem by the use of a prospective diary. Since phantom sensations and phantom pain do occur in adolescent amputees, this population warrants further investigation, especially in determining how to prevent and treat these phenomena.

## Conclusions

PLP was found in 29% of the amputees in China, which had markedly affected the lives of patients. We found that pre-amputation pain and the use of postoperative epidural analgesia which aggravates the sudden deafferentation of the amputated extremities were risk factors for the development of phantom limb pain. This finding suggests that prevention of pre-amputation pain and sudden post-amputation deafferentation should be recommended to the amputees.

## Abbreviations

CNY: Chinese Yuan
ICC: Intraclass correlation
NRS: Numeric rate scale
PLP: Phantom limb pain
PLS: Phantom limb sensations
